# Intracellular Survival and Pathogenicity Modulation of *Salmonella* Lon, CpxR, and RfaL Mutants Used as Live Bacterial Vectors under Abiotic Stress, Unveiling the Link between Stress Response and Virulence in Epithelial Cells

**DOI:** 10.3390/ijms25169056

**Published:** 2024-08-21

**Authors:** Perumalraja Kirthika, Amal Senevirathne, Sungwoo Park, Ram Prasad Aganja, In-Shik Kim, Hyun-Jin Tae, John Hwa Lee

**Affiliations:** 1 College of Veterinary Medicine, Jeonbuk National University, Iksan 54596, Republic of Korea; pkirthika@mayo.edu (P.K.); amal.senevirathne@jbnu.ac.kr (A.S.); ramaganja@jbnu.ac.kr (R.P.A.); 2 College of Veterinary Medicine and Institute of Animal Transplantation, Jeonbuk National University, Iksan 54596, Republic of Korea; iskim@jbnu.ac.kr (I.-S.K.); hjtae@jbnu.ac.kr (H.-J.T.)

**Keywords:** *Salmonella* Typhimurium, intracellular survival, abiotic stress, NF-kB, chemokines, cell cycle arrest

## Abstract

In the current study, two *Salmonella* Typhimurium strains, JOL 912 and JOL 1800, were engineered from the wild-type JOL 401 strain through in-frame deletions of the *lon* and *cpxR* genes, with JOL 1800 also lacking *rfaL*. These deletions significantly attenuated the strains, impairing their intracellular survival and creating unique immunological profiles. This study investigates the response of these strains to various abiotic stress conditions commonly experienced in vivo, including temperature, acidity, osmotic, and oxidative stress. Notably, cold stress induced a non-significant trend towards increased invasion by *Salmonella* compared to other stressors. Despite the observed attenuation, no significant alterations in entry mechanisms (trigger vs. zipper) were noted between these strains, although variations were evident depending on the host cell type. Both strains effectively localized within the cytoplasm, demonstrating their ability to invade and interact with the intracellular environment. Immunologically, JOL 912 elicited a robust response, marked by substantial activation of nuclear factor kappa B (NF-kB), and chemokines, interleukin 8 (CXCL 8) and interleukin 10 (CXCL 10), comparable to the wild-type JOL 401 (over a fourfold increase compared to JOL 1800). In contrast, JOL 1800 exhibited a minimal immune response. Additionally, these attenuations influenced the expression of cyclins D1 and B1 and caspases 3 and 7, indicating cell cycle arrest at the G2/M phase and promotion of the G0/G1 to S phase transition, alongside apoptosis in infected cells. These findings provide valuable insights into the mechanisms governing the association, internalization, and survival of *Salmonella* mutants, enhancing our understanding of their regulatory effects on host cell physiology.

## 1. Introduction

Live bacterial vectors, also known as LBVs, are interesting and efficient tools available as alternative methods of vaccine delivery [1]. Over half a century, many pathogen species have been tested and recognized for their impressive ability to be utilized in vaccine delivery and immune therapeutic applications. Some of these species are *Escherichia coli* [2], *Salmonella* Typhimurium [3], *Listeria monocytogenes* [4,5], *Shigella flexneri* [6], *Vibrio cholera* [7], *Vibrio Harvey* [8], and *Lactobacillus species* [9]. These species have been utilized in pre-clinical and clinical studies with promising outcomes [10]. The attenuation of pathogenic bacteria is derived in multiple ways, such as by chemical, physical, radiation, and genetic methods [11]. Unlike inactivated or killed vaccines, live attenuated vaccines conserve strong potential to induce both systemic humoral and cell-mediated immune responses, often bringing life-long protection owing to immunological memory [12,13]. Strong immune responses can be anticipated from live attenuated vaccines because of their close resemblance to a natural infection. Using genetic engineering technology, LBVs can be manipulated in various ways to deliver heterologous antigens, thus protecting against various infectious diseases, including bacterial and viral diseases.

Among several suitable bacterial pathogens for the development of LBVs, *Salmonella* is one of the prominent and well-curated organisms for investigation [14]. Genetic modifications of the *Salmonella* genome are commonly used, and several auxotrophic *Salmonella* strains have been investigated with improved safety. The construction of *aroA* knocked-out mutants occurred about 30 years ago and is still widely used [15]. Owing to the advancement of genetic engineering technology, and sequencing platforms, in-frame deletion of open reading frames can now be achieved with high precision [16]. The choice of target genes can be based on various research perspectives. Several curated attenuation markers can be highlighted as genes related to bacterial survival and replication in host cells such as *lon* [17], genes related to nucleic acid synthesis (*purI*, *guaBA*, and *galE*) [18,19,20], genes related to the microbial DNA repair mechanism (*recA* and *recBC*) [21], synthesis of outer membrane proteins (o*pmC*, ompF) [22], etc.

For vaccine-related applications, we constructed *Salmonella* Typhimurium attenuated strains by deleting *lon* and *cpxR* (JOL 912) [23], and *lon*, *cpxR*, and *rfaL* (JOL 1800) [24] for particular purposes. These strains are immunologically potent, yet significantly attenuated without chronic survival in macrophages and in host tissues. The two strains were exclusively used in vaccine investigation for the delivery of heterologous antigens; thus, as an auxotrophic marker, aspartate-semialdehyde dehydrogenase (*asd*) gene was deleted in both strains. The growth of *asd* mutant strains was assured by supplementation of diaminopimelic acid (DAP) or by genomic complementation of *asd* gene via a therapeutic plasmid. The *lon* gene encodes for evolutionarily conserved stress protein and is found in the cytoplasm of prokaryotes and a mitochondrial matrix protein in eukaryotes. It plays a vital role in intracellular survival and regulates the bacterial invasion in epithelial cells, thus playing an important role in systemic infection [25]. *cpxR* is the response regulator part of a two-component signal transduction pathway where *cpxA* is the sensor kinase. *CpxR/A* is one of the three main extracytoplasmic stress response (ESR) pathways in *Salmonella* [17]. This pathway helps the bacteria alter its envelope physiology during environmental stress and survive the insult. It plays a key role in pilus assembly, motility, and chemotaxis, and regulates the type III secretion system and biofilm formation. Thereby, *cpxR* is involved in the invasion of epithelial cells [26].

Although the deletion of the two major bacterial virulence-regulating genes *lon* and *cpxR* contributed to the attenuation of the bacteria, the deletion of *rfaL* further reduces virulence. This gene is functionally homologous to *rfbT* and *waaL* and involved in the attachment of O-antigen to the core. *rfaL* gene knockout mutants of ST seldom produce smooth lipopolysaccharide (LPS) but synthesize complete O-antigen side chains attached to alanyl-cardiolipin (ACL) and rough LPS. This shows that it may encode a part of O-antigen ligase that participates in the transfer of O-antigen to an LPS acceptor. With respect to LPS structure, JOL 912 and JOL 1800 are similar to JOL 401 (wild type; WT) except that they have a truncation of cytoplasmic, extracytoplasmic, and cell wall portions. Both the mutants are a feasible option as LBVs and are suitable for stimulating the optimal protective immunity to homologous or heterologous antigens by oral, intranasal, and intramuscular routes of administration by manipulating dose regimes specific for each mutant strain. The triple and quadruple mutants have fared well as LBVs in experimental animals maintained under normal physiological conditions. The stress adaptation of JOL 912 and JOL 1800 are yet to be elucidated. Their ability to adapt to a diverse range of abiotic stresses may aid in their survival, colonization, and virulence in the host.

Being live attenuated vaccines, they are ideally inoculated via the oral route; therefore, bacteria strains should be persistent with virulence, sufficient enough to colonize and invade the intestinal mucosa [27,28]. On the other hand, within the oral route, bacteria species are exposed to various environmental stress conditions, such as extremely acidic environments, osmotic stress conditions, temperature shocks, and oxidative stress conditions. A bacterium such as *Salmonella* uses various strategies to mitigate such stress conditions while modulating its virulence to survive in such adverse conditions. It is imperative to know the exact behavior of LBVs under these stress conditions as a means to understand their virulence phenotypes to ensure safety. Attenuated mutants can behave significantly differently from wild-type strains; hence, to fulfill this objective, in the current study, we investigated the effect of individual abiotic stress on the interaction between ST mutants and host cells compared to their wild-type counterpart JOL 401’s behavior. We interpreted the effect of acid stress (pH 3.5, 5 h), cold stress (4 °C, 5 h), oxidative stress (1 mM H_2_O_2_), and osmotic stress (5% wt/vol) NaCl on the ability to interact and invade HeLa and HepG2 cells. The comparative analysis of different immune-regulating key cytokines, cell cycle-regulating cyclins, and apoptotic genes’ expression in cells infected with the mutants JOL 912 and 1800 was carried out as a comparative study against the wild-type JOL 401 strain. This allowed for a better understanding of the actual behavior of these attenuations in determining favorable features as an LBV strain.

## 2. Results

### 2.1. ST Mutants (JOL 912 and JOL 1800) Exposed to Abiotic Stress Remain Invasive in Both Eukaryotic Cell Lines

**Figure 1:**
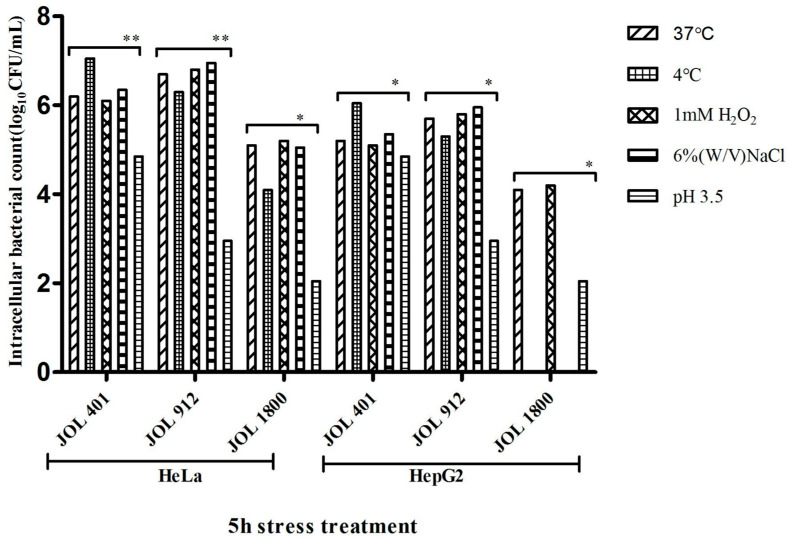
Invasion and intracellular replication in HeLa and HepG2 cells by *Salmonella* Typhimurium, JOL 401, JOL 912, and JOL 1800. Cells were infected with *Salmonella* WT, and mutants subjected to cold, oxidative, osmotic, and acidic stress conditions at an MOI of 5, and afterward, medium without gentamicin was added for 1 h. Next, this medium was replaced by a medium containing gentamicin at 10 µg/mL. Intracellular replication was calculated by taking the ratio of CFU recovered 8 h post-infection (hpi) to 1 hpi. Mean values and standard deviations (SD) obtained from three independent experiments are demonstrated. The level of significance was indicated as * *p* < 0.05; ** *p* < 0.01.

Environmental stress confers a significant effect on the bacterial physiology and thus virulence of each strain. Such modulations can affect the virulence traits of a bacterium, such as adhesion, invasion, and intracellular survival. In this study, we characterized *Salmonella* WT strain JOL 401 and two attenuated strains JOL 912 and JOL 1800, which are employed in immunization investigations. JOL 912 is a smooth strain with a complete lipopolysaccharide structure (LPS) whereas JOL 1800 contains a truncated version of LPS, owing to lack of *rfaL* gene (O-antigen ligase). We could practically observe that the level of protection induced by JOL 912 was superior to that of JOL 1800, while JOL 1800 was significantly safer than the other strain. In this study, we observed their virulence behavior under induced stress conditions (temperature, acidic, oxidative, and osmotic) that these bacteria strains normally experience in the in vivo environment. After the stress treatment, the invasive ability of both the mutants was studied using a gentamicin protection assay on HeLa and HepG2 cells and compared against the WT strain JOL 401. Gene knockout did not deter bacterial adhesion and invasion, as no significant difference was observed in the total number of adhered cells between the mutants and the wild-type strain regardless of their ability to proliferate within infected cells. Among the treatments in both cell lines, the WT strain JOL 401 subjected to cold stress showed significantly higher (*p* < 0.05) intracellular replication compared to the other treatment whereas the invasion was significantly low in cells exposed to osmotic stress (5% (wt/vol) NaCl) and further decreased with oxidative stress (1 mM H_2_O_2_) (Figure 1). The lowest bacterial counts were observed with acid-treated cells. Altered patterns of intracellular replication were observed with JOL 912 and JOL 1800; however, it was a gradually reducing pattern compared to the wild-type JOL 401 strain. It was evident that the *Salmonella* invasiveness is higher in HeLa cells compared to HepG2 cells. We also noticed that JOL 1800 exposed to cold shock and osmotic stress conditions failed to proliferate within HepG2 cells (Figure 1).

### 2.2. Fenton Reaction: Free Radical Production Is Dependent on Gene Knockout and pH

**Figure 2:**
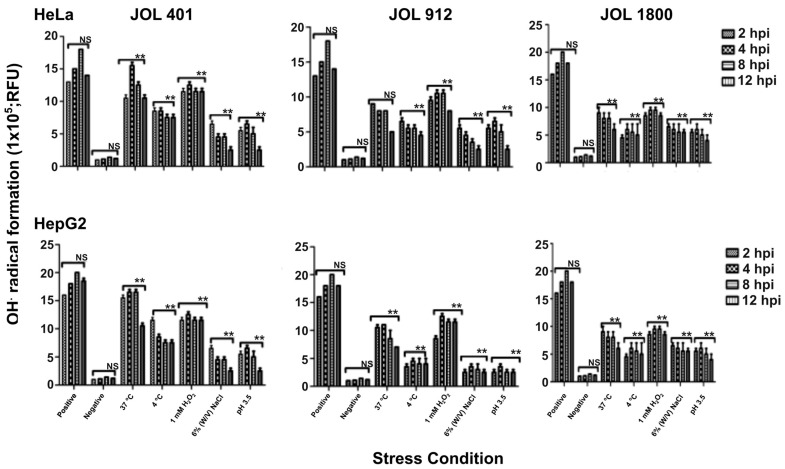
Kinetics of Fenton reaction in *Salmonella* Typhimurium-, JOL 401-, JOL 912-, and JOL 1800-stimulated HeLa and HepG2 Cells. Results are expressed RLUs in ST-stimulated Cells compared to unstimulated cells. Cells treated with 10 µM CuCl_2_ and 100 µM H_2_O_2_ at 37 °C for 1 h were served as a positive control, whereas non-treated cells served as a negative control. Mean values and standard deviations (SDs) obtained from three independent experiments are demonstrated. The levels of significance are indicated as ** *p* < 0.05. “hpi” stands for hours post-infection, “NS” stands for non-significant.

In the Fenton reaction, Fe (II) ions react with H_2_O_2_ to produce hydroxyl radicals (OH) [29] that cause DNA damage and ultimately cell death. The Fenton reaction is of prime importance in the eradication of bacterial infections and toxic OH· and also induces tumor cell apoptosis [30]. In both HeLa and HepG2 cell lines, the WT strain grown at 37 °C resulted in higher OH· radical generation, followed by 1 mM H_2_O_2_ treatment and pH 3.5 exposure. In JOL 912 mutants, the highest OH· radical generation resulted under 1 mM H_2_O_2_ oxidative stress followed by pH 3.5 acidic exposure (Figure 2). The generation of OH· radicals by both JOL 912 and JOL 1800 mutants under normal physiological temperature conditions was lower compared to the WT JOL 401 strain, further confirming their respective attenuation conferred by the deletion of virulence genes. Under each condition, peak levels of free radical generation were observed between 2 and 8 h post-infection (hpi) and then resulted in a dwindling pattern towards 12 hpi.

### 2.3. Invasion of HeLa and HepG2 by ST Causes Actin Rearrangement

**Figure 3:**
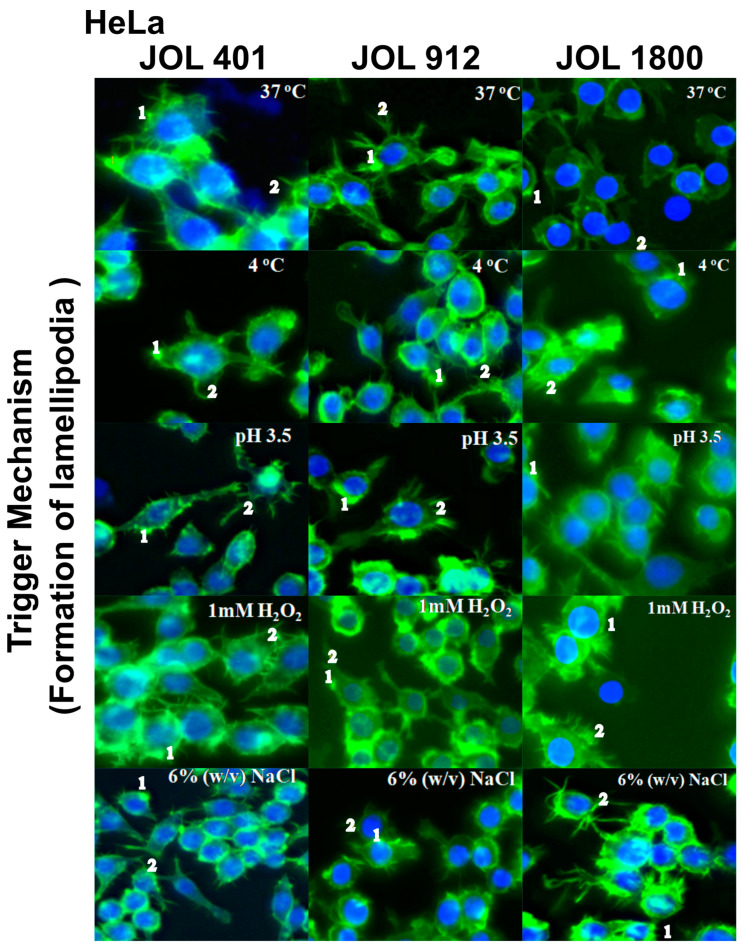
Trigger mechanisms used by *Salmonella* to HeLa cells. HeLa cells were infected with WT JOL 401 and mutant strains JOL 912 and JOL 1800, subjected to various stress conditions for 90 min, fixed, and labeled with phalloidin to visualize F-actin (green in merged images), and DAPI was used to label the nucleus (blue color). Images are micrographs of representative infected cells. 1. Accumulation of polymerized actin. 2. Membrane rearrangement and cellular protrusions are depicted.

**Figure 4:**
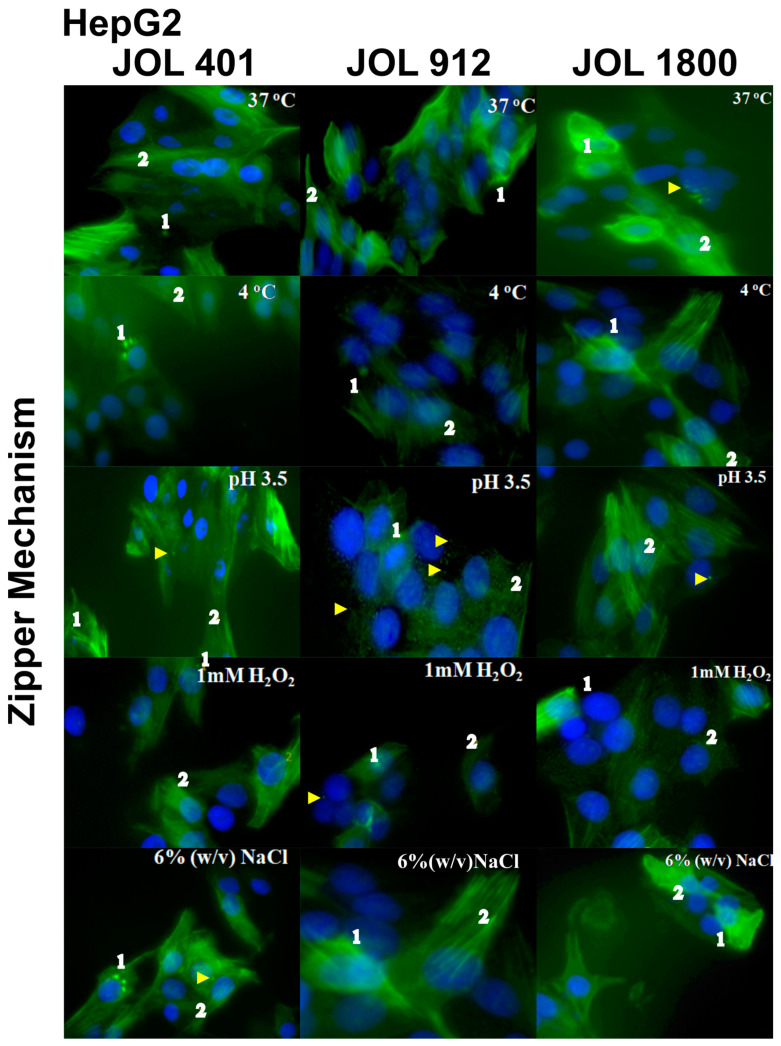
Zipper mechanisms used by *Salmonella* to HepG2 cells. HepG2 cells were infected with WT JOL 401 and mutant strains JOL 912 and JOL 1800, subjected to various stress conditions for 90 min, fixed, and labeled with phalloidin to visualize F-actin (green in merged images), and DAPI was used to label the nucleus (blue color). Images are micrographs of representative infected cells. 1. Accumulation of polymerized actin. 2. Sites of milder actin remodeling. Yellow arrows indicate intracellular *Salmonella*.

Invasive pathogens possess the ability to manipulate the actin cytoskeleton [31]. Investigations conducted in HeLa and HepG2 cells revealed a distinct actin rearrangement irrespective of the treatment. In HeLa cells, accumulation of polymerized actin was evident (Figure 3). It was also known that the invasion of *Salmonella* involves either a trigger or a zipper mechanism mediated by the T3SS-1 [32] or the invasin *Rck*, respectively [33]. Another outer membrane protein, *PagN*, was also implicated in the invasion [34]. An intense cytoskeletal remodeling was prominent in HeLa whereas HepG2 showed a little change in the cytoskeleton (Figure 4). To further ensure that these observations were not time-dependent, observations were conducted at different post-infection times at 15, 30, 45, 60, 90, 120, and 150 min however, no significant difference could be observed between wild-type JOL 401 and mutant strains.

### 2.4. ST Localization and Intracellular Spread

**Figure 5:**
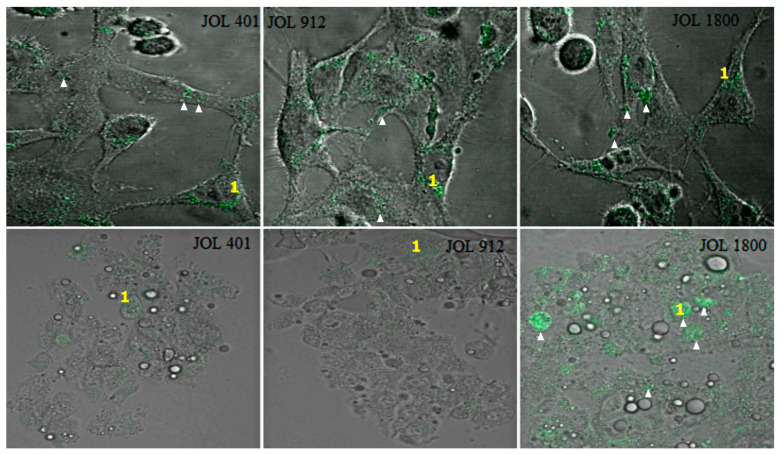
Localization of *Salmonella* in HepG2 cells. The mechanisms that direct the invading bacterium to follow the cytosolic or intra-vacuolar “pathway” remain poorly understood. In vitro studies show a predominance of either the cytosolic or the intra-vacuolar population depending on the host cell type invaded by the pathogen. 1. Sites of bacterial accumulations. White arrows indicate their spread within the cytoplasm, either scattered or in clusters.

The stress treatment did not show any varied changes in the interaction at the host cell plasma membrane but seemed to be host-cell-specific. Consequently, for the localization assay, we studied ST interaction with HepG2 cells after growing bacteria at 37 °C. The confocal images showed that all three ST, JOL 401, JOL 912, and JOL 1800, localized in the cytosol. The ability of the bacteria to replicate in *Salmonella*-containing vacuole (SCV) and cytosol varied among the mutants. The WT JOL4 01 appeared scattered in the infected cell; however, JOL 912 and JOL 1800 showed clustered localization, which might be within SCVs. It was evident that JOL 1800 remained in the SCV for a longer time (3 hpi) while JOL 12 lysed the vacuole and reached the cytosol within 90 min (Figure 5).

### 2.5. Expression of Cytokine, Cyclin, and Caspase Transcripts

**Figure 6:**
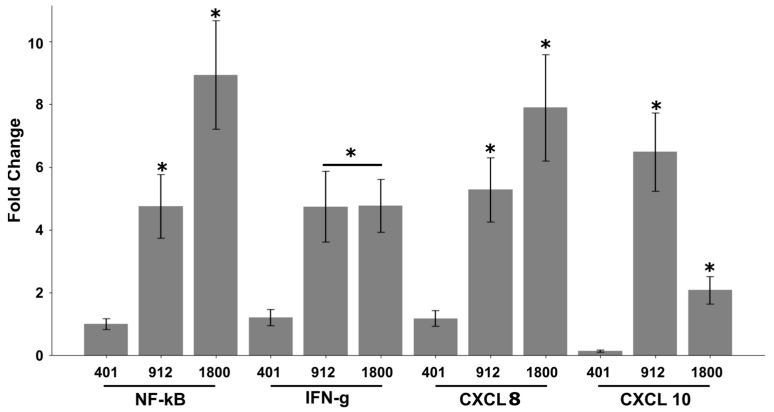
Modulation of cytokines and chemokines owing to *Salmonella* WT and mutant infection. Expression of NF-κB, IFN-γ, CXCL 8, and CXCL 10 messenger RNA (mRNA) expression in *Salmonella* Typhimurium-, JOL 401-, JOL 912-, and JOL 1800-stimulated HepG2 Cells after 12 h incubation. Results are expressed as mean fold change in cytokine transcripts in ST-stimulated cells to unstimulated cells. Mean values and standard deviations (SDs) obtained from two biological replicates are demonstrated. The level of significance is indicated as * *p* < 0.05.

Activation of NF-kB is essential for mounting a proper inflammatory response against invading bacteria [35]. In this study, a comparison of JOL401, the WT strain, against two mutants, JOL 912 and 1800, revealed an 8.9-fold increase in expression in JOL 401-treated cells, higher than that of JOL 912, and treated HepG2 cells in 12 h (4.7-fold) (Figure 6). The induction of NF-kB by the JOL1800 mutant was low compared to the wild-type strain. The expression levels of IFN-γ were also lowest in JOL 1800 in the 12 h incubation period (1.2-fold), whereas JOL 912 and WT JOL 401 strains obtained comparable results (4.74- and 4.77-fold). The expression of chemokine marker CXCL 8, which plays a vital role in the chemotaxis of immune cells to the site of infection, was also high in JOL 401 and JOL 912 (7.8- and 5.2-fold), whereas expression in JOL 1800 was only 1.2-fold [36] (Figure 6). The CXCL 10 chemokine, also known as interferon gamma-induced protein 10 (IP-10) showed a slightly different pattern, where JOL 912 yielded the highest expression, 6.4-fold, whereas the WT strain received a 2-fold increase while JOL 1800 obtained the lowest expression, 0.14-fold. These data suggest that the mutant JOL 912 is a more potent LBV strain in immune induction, in comparison to JOL 1800, potentially owing to the deletion of three virulence-related genes in the JOL 1800 genome. When we assessed the HeLa cells for NF-kB expression, we could not see any appreciable induction, possibly owing to a lack of accessory proteins such as MD-2, which is essential for mounting TLR4-induced inflammatory responses [37,38].

**Figure 7:**
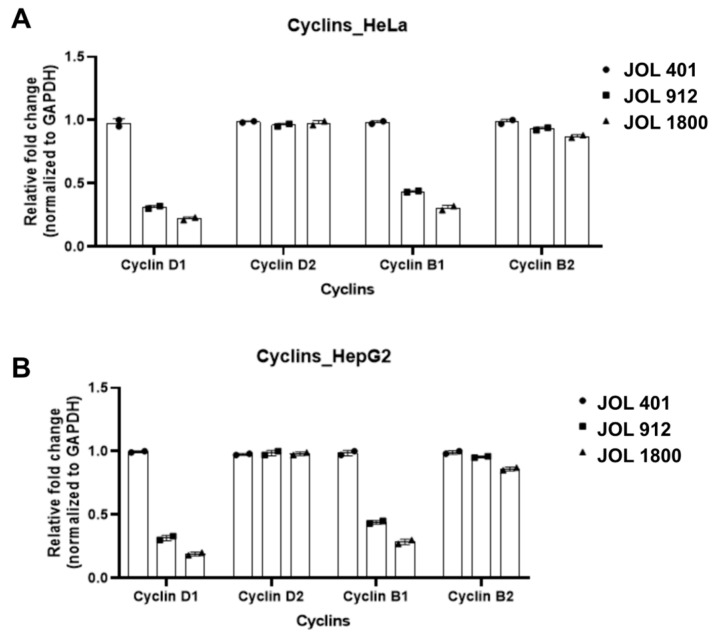
Expression of cyclin D1, cyclin D2, cyclin B1, and cyclin B2 messenger RNA (mRNA) expression in *Salmonella* Typhimurium- and JOL 401-stimulated (**A**) HeLa and (**B**) HepG2 Cells. Results are expressed as mean fold change in cytokine transcripts in ST-stimulated cells at various hpi compared to unstimulated cells. Mean values and standard deviations (SDs) obtained from two biological replicates are demonstrated. The level of significance is determined at *p* < 0.05.

**Figure 8:**
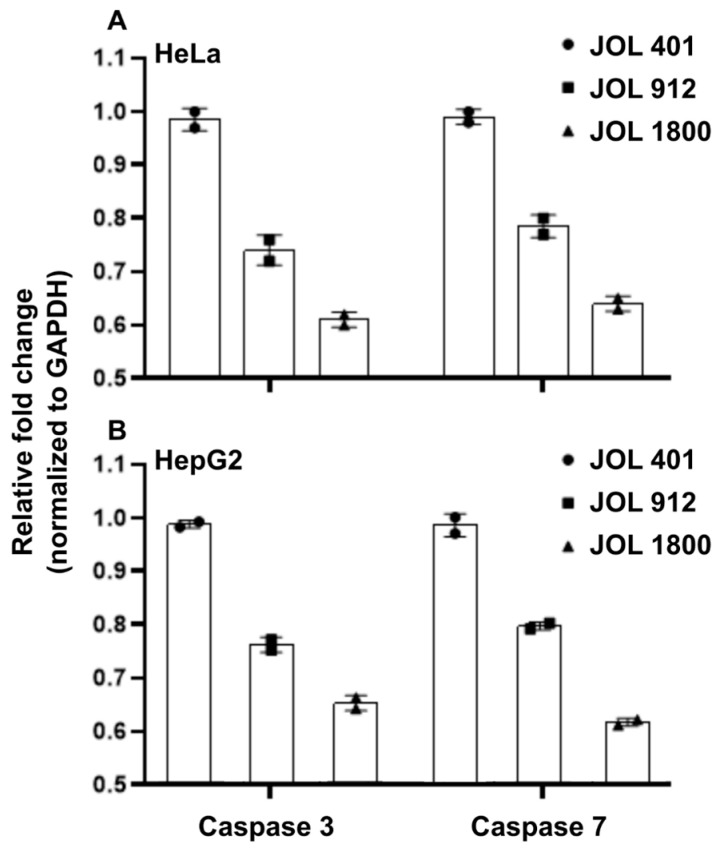
Expression of caspase 3 and caspase 7 messenger RNA (mRNA) expression in *Salmonella* Typhimurium- and JOL 401-stimulated (**A**) HeLa cells and (**B**) HepG2 Cells. Results are expressed as mean fold change in gene transcripts in ST-stimulated cells compared to unstimulated cells. Mean values and standard deviations (SD) obtained from three independent experiments are demonstrated. The level of significance was determined at *p* < 0.05.

The relative expression of cyclin D1 decreased after 12 hpi in HeLa and HepG2 cells infected with bacterial mutants compared to the WT strain (Figure 7A,B). JOL 1800-infected cells recorded the least amount of cyclin D1 mRNA expression compared to JOL 912 and JOL 401 (*p* < 0.05). The levels of cyclin B1 were also significantly low (*p* < 0.05; Figure 7) for HeLa and HepG2 infected with JOL 1800. Expression of cyclin D2 and B2 relatively maintained among all three strains without much of a difference in mutants. Furthermore, the expression of caspases was compared in HeLa and HepG2 after *Salmonella* WT JOL 401 and mutant JOL 912 and 1800 infection, which revealed caspase 3 and 7 expression and showed a reducing pattern in JOL 912- and JOL1800-treated cells, compared to the WT strain (Figure 8A). The expression of caspases were following a similar pattern in both HeLa and HepG2 cells, whose expression was highest in ST WT JOL 401-treated cells, while showing a dwindling pattern towards JOL 912 and JOL 1800 (Figure 8A,B).

### 2.6. Effect on Cell Cycle Kinetics Demarcates G2/M Arrest

**Figure 9:**
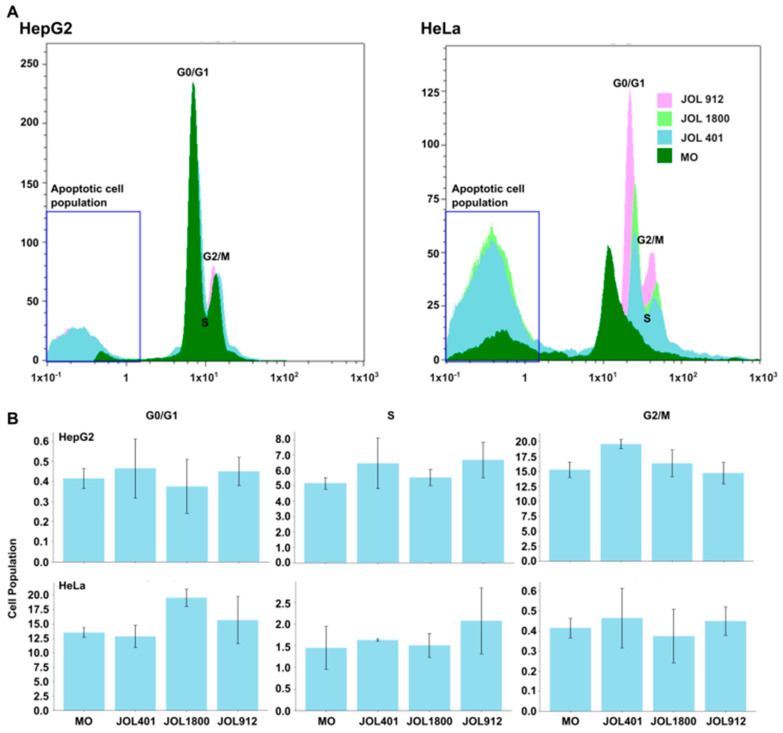
Cell cycle analysis by propidium iodide staining. (**A**) HepG2 and HeLa cells were infected with JOL 401, JOL 912, and JOL 1800. The effect of infection on the cell cycle was investigated using the PI staining method. Cells were gated to eliminate the dead cell population, and the remaining cells were assessed for red fluorescence. A histogram was prepared and the effect against each treatment relative to G0/G1, S, and G2/M phases. Encaged section demarcates induced apoptotic cell population. (**B**) Proportions of each G0/G1, S, and G2/M cell population were quantified and presented as a bar graph. Mean values and standard deviations (SDs) obtained from two biological replicates are demonstrated. The level of significance is determined at *p* < 0.05.

Furthermore, cell cycle analysis was conducted by the propidium iodide staining method, which resulted in an increase in the G2/M phase by JOL 401, whereas JOL 912 demonstrated an increased accumulation of the S phase (Figure 9). Not much of an effect was observed for the G0/G1 phase. Interestingly, an increase in apoptotic cell population was evident for all three *Salmonella* strains in both HeLa and HepG2 cells compared to the treatment control with media alone. The infection of *Salmonella* can influence the host cell cycle, as these bacteria attempt to slow down the cell cycle in facilitating their invasion and replication. In our study, we observed an accumulation of cells at the G2/M phase by wild-type *Salmonella* for both HeLa and HepG2 cells, however with a significant accumulation of cells in HepG2 cells (17.5%). The same effect was lower in mutant strains JOL 912 and 1800. On HeLa cells, more cells were accumulated in the G0/G1 phase for mutant strains JOL 1800 and 912. Previous investigations also reported that the *Salmonella* strains are capable of selectively inhibiting the G2/M phase by the potential interaction of effector proteins with cellular epidermal growth factor receptors (EGFRs) [39,40,41].

## 3. Discussion

The introduction of specific in-frame deletions of essential virulence factors into the *Salmonella* genome has been widely used to generate live vaccine carriers. These LBVs possess the ability to invade the enterocytes and stimulate a memory B- and T-cell immune response [13]. Regarding the ST mutants, JOL 912, being *cpxR* and *Lon* deficient, and JOL 1800, being *cpxR, Lon,* and *rfaL* deficient, are smooth and rough strains, respectively. The two bacteria strains are significantly attenuated without being able to proliferate or survive in the intracellular environment due to enhanced susceptibility to oxidative damage inflicted by the host cell [25]. These two strains were successfully utilized in immunization experiments to deliver heterologous antigens against various bacterial and viral diseases [42,43]. Both these LBVs possess the ability to elicit an immune response but are avirulent as compared to the WT JOL 401 strain. When LBVs are exposed to an in vivo environment, these attenuated bacterial pathogens are naturally exposed to various stress conditions during the invasion and colonization leading to an infection. During passage through the gut, gastrointestinal pathogens are exposed to pH and osmotic stresses, and they experience oxidative stress during invasion into cells. Herein, we pre-exposed bacteria to various stress conditions and examined how they would interact with epithelial cells, using HeLa and HepG2 as a model for epithelial invasion. Despite significant attenuations caused by *lon, cpxR,* and *rfaL* gene deletions, these two mutant strains did not show significant compromise in invasion capability, as they might have learned new mechanisms to invade host cells [44]. Another possibility can be the deletion of the *lon* gene, which encodes a protease that acts as a negative regulator of virulence-related genes, and modulates enhanced virulence phenotype, counteracting the lack of three virulence genes [45], especially SPI-I genes. Among cold, acidic, osmotic, and oxidative stress conditions, a significant enhancement in invasion was observed for cold stress conditions in the ST WT strain [46]. On the other hand, a cold-induced hypervirulence phenotype has been reported with other bacteria species too, such as *Cronobacter sakazakii* [47].

Before invasion, the interaction of bacteria with host cells causes remodeling of the membrane cytoskeleton [48]. Based on the actin remodeling and morphological changes, this mechanism is classified into the zipper and trigger phagocytosis [49]. *Salmonella* species have the distinction to induce both zipper and trigger mechanisms based on the host cell type [33]. The phalloidin staining showed that the actin rearrangement was distinct for both HeLa and HepG2. In HeLa, an intense actin remodeling and formation of pseudopodia-like structures were observed. The trigger mechanism of invasion is evident in HeLa cells, and this may be attributed to T3SS-1, a needle-like structure encoded by *Salmonella* pathogenicity island 1 (SPI-1) that injects bacterial protein into cell cytoplasm. This leads to intracellular cascades that cause intense actin rearrangement and the internalization of bacteria, which promotes the pathogen’s engulfing of the host cell [50]. In HepG2 cells, the local actin accumulation is evident, which leads only to minor cytoskeletal actin rearrangements and tight membrane extensions. Unlike the trigger mechanism, in the zipper mechanism, the interaction of a bacterial ligand with a host cell receptor is crucial for invasion. We believe the zipper mechanism in the HepG2 is due to the over-expression of epidermal growth factor receptor (EGFR) in this kind of cancer cell [51], which is the receptor for one of the outer membrane proteins, *Rck* [40]. Along with *PagN*, *Rck* actively mediates the zipper cascade of invasion and internalization of the bacteria. Similar to cytoskeleton remodeling, the localization of the bacteria (JOL 401, 912, and 1800; exposed to various stresses) was host-cell-specific. Confocal microscopy showed approximately 10–15% of the intra-vacuolar bacteria gain access to the cytosol where hyper-replication occurs reaching up to >100 individuals per infected HeLa cell [52]. In HepG2, the ability of the bacteria to leave the intracellular vacuoles seemed to be less than that in HeLa, and no hyper-replication was observed.

Host defenses leverage direct and indirect strategies to eliminate the invading bacteria. The bacteria, on the other hand, make efforts to bypass the host tactics to establish the infection. Some cascades favor the host cell, such as the production of hydroxyl radicals and secretion of cytokines; some help the bacteria, such as cell cycle arrest; while some other features help both the host and the pathogen, for instance, the activation of caspases. The cold-treated WT and mutant infection in HeLa and HepG2 produced lower levels of hydroxyl radical, which we assume will circumvent the host defense and increase the pathogenicity of the bacteria. The acid-treated bacteria (all three) enhanced the hydroxyl radical production, and this may be attributed to bacterial free radicals that are synthesized before the infection due to the falling pH.

During an infection, pro-inflammatory responses are induced by bacterial molecules such as LPS, pili, flagellin, etc., also known as pathogen-associated molecular patterns (PAMPs) [53]. These molecules are readily detected by host Toll-like receptors, which induces a cascade of downstream signaling pathways, leading to the activation of Nf-kB-mediated defense responses. In this respect, *Salmonella* WT JOL 401 and mutant strains JOL 912 and JOL 1800 are fairly similar, without significant alterations. The mutant strains are immunologically well-potent in eliciting immune responses. In this study, we examined the expression of cytokines NF-kB and IFN-γ and chemokines CXCL 8 and CXCL 10 using a HepG2 cell-derived qRT-PCR assay. As anticipated, JOL401 was a potent inducer for all candidate genes, whose expression levels were closely followed by the JOL 912 strain. However, the induction of these markers was comparatively less in JOL 1800, whose only difference was in the lack of an O-antigen component in the LPS structure, compared to JOL 912. This observation indicates the significance of O-antigen in LPS structure in modulating inflammatory responses, though it is not a key player in TLR4 activation. The O-antigen is known to evade host-immune defense during early infection; thus, lack of this essential component may escalate susceptibility to host defense by limiting JOL 1800 critical cell numbers [54]. Among various cyclins involved in cell cycle progression, cyclin B1 plays a crucial role in arrest at the G2/M phase [55]. Previous studies also reported that *Salmonella* can affect G2/M phase cell cycle arrest [56]. The cell cycle halt in this phase is crucial for *Salmonella’s* intracellular proliferation [56]. It also increases the transition from the G1 to the S phase of the cell cycle. Our analysis of cyclin D1, D2, B1, and B2 revealed that *Salmonella* infection in HeLa and HepG2 only affects D1 and B1. Expression of these cyclins was lower in mutants than in the wild-type strain (Figure 7). Furthermore, only a slight increase in the levels of caspases-3 mRNA levels in cells infected with JOL 401 exposed to the stress conditions shows that the genes deleted (lon, cpxR, and rfaL) act as a pro-survival factor by preventing the early-onset apoptosis in epithelial cells.

These observations are partially supported by our observations in PI-based cell cycle analysis, which revealed cell cycle arrest in the G2/M phase (increase in cell number), while the marginal increase in the S phase is possible due to increased transition from G0/G1 to S phase. Comparing mutant strains revealed almost non-significant differences, however with a prominent increase in apoptotic cell populations (Figure 9).

Results of the current study provide evidence for the dynamic interaction of *Salmonella* JOL 401 WT strain and its mutants JOL 912 and 1800 based on environmental stress conditions and host-cell type demonstrating their tailored fashion of infection under various conditions. It also reveals that despite significant attenuations, *Salmonella is* still capable of modulating the host immune environment in favor of its survival under in vivo conditions. The immunological differences between JOL 912 and JOL 1800 suggest that despite the elicitation of antibody responses and host immune evasion during early phases, *Salmonella*’s O-antigen plays a crucial role in mounting proper inflammatory responses. The insight gathered by this study helps us to better comprehend the behavior of LBVs JOL 912 and JOL 1800 during infection and how they elicit specific immune functions owing to their built-in mutations.

## 4. Materials and Methods

### 4.1. Bacterial Strains

Bacterial strains used in this study are listed in Table 1. The smooth mutant, JOL 912, was constructed from a JOL 911 (*cpxR* and *lon* genes deleted) strain with an allelic exchange method [57]. A rough auxotrophic mutant, JOL 1800, was created from JOL 912 by the deletion of *rfaL* by the gene red lambda recombineering approach [24]. In both cases, the asd+ plasmid vector creates a balanced lethal complementation between the host and the plasmid construct. A fresh vial of culture was transferred to LB broth and incubated for 12–16 h (37 °C, 220 rpm). The bacteria in the log phase were used as their susceptibility to stress conditions is better than those in the stationary phase.

### 4.2. Cell Culture

The human epithelial cell HeLa and HepG2 were procured from the American Type Culture Collection (Manassas, VA, USA) and were maintained in Dulbecco’s Modified Eagle Medium (DMEM), high glucose (Lonza, Walkersville, MD, USA) containing 10% fetal bovine serum (FBS, Lonza), 100 U/mL penicillin, 100 I.U./mL streptomycin, and L-glutamine (0.3 mg/mL).

### 4.3. Bacterial Growth under Stress Conditions

Bacteria were grown to log phase and subjected to various stress conditions according to Shah et al., with modifications [58]. Briefly, the bacterial cells were pelleted by centrifuging at 3500× *g* for 15 min and re-suspended (~10^8^ CFU/mL) into LB at pH 3.5 (pre-warmed to and adjusted with hydrochloric acid; acid stress), in pre-chilled LB (at 4 °C; cold stress), in LB with 1 mM H_2_O_2_ (pre-warmed to 37 °C; oxidative stress), and in LB with 5% (wt/vol) NaCl (pre-warmed to 37 °C; osmotic stress), and grown for 5 h at 220 rpm. The OD_600_ was read, bacterial culture was centrifuged at 3500× *g* for 15 min and re-suspended in 1 mL of phosphate-buffered saline (PBS). This suspension was diluted in DMEM (no antibiotics) at a concentration of 5 × 10^6^ CFU/mL. Non-stressed ST culture in DMEM maintained at 37 °C served as negative control.

### 4.4. Bacterial Invasion and Intracellular Replication

The invasion assay was performed according to Wu et al., (2014) with modifications [59]. Briefly, the HeLa and HepG2 cells were cultured in Dulbecco’s Modified Eagle Medium (DMEM) supplemented with 10% fetal calf serum, 2 mM L-glutamine, and 100 μg/mL penicillin and streptomycin. When the cells reached a 60–80% confluence in the flask, they were harvested and re-suspended at a concentration of 2.5 × 10^5^ live cells/mL in fresh DMEM and plated in a 12-well plate. Each well received 1 mL of DMEM cell culture medium containing the aforementioned concentration. Cells were incubated at 37 °C in a 5% CO_2_ incubator overnight. The log phase culture of the bacteria (JOL 401, 912, and 1800 at 37 °C and exposed to various stresses). The culture was diluted to 2 × 10^7^ CFU/mL in DMEM without antibiotics. Each well of the tissue culture plate was washed once with 1 mL of 1× PBS buffer. After the removal of PBS, bacterial suspension was added to each well to achieve a multiplicity of infection (MOI) of 5:1 in complete media. The plates were centrifuged at 200× *g* for 5 min to synchronize the interaction between bacteria and monolayer cells and incubated at 37 °C in a CO_2_ (5%) incubator to allow attachment for 1 h. Wells were washed 3 times with PBS and added with 1 mL of DMEM containing 100 μg/mL gentamicin to kill extracellular bacteria. Cell monolayers were further incubated for an additional 1 h at 37 °C. The cells were again washed three times with PBS and supplemented with complete media containing 10 μg/mL gentamycin. Cells were further incubated, and lysis was conducted at 1 and 8 h post-infection (hpi) by adding 500 µL of lysis buffer (PBS with 1% Triton X-100 and 0.1% SDS). Aliquots of serial dilutions made from each well were spread onto LB plates and incubated overnight to determine the number of invading bacteria. The ratio between bacterial numbers enumerated at 8 h and 1 h was considered as bacterial intracellular replication.

### 4.5. Hydroxyl Radical (OH·) Measurement

OH· measurement was performed following the manufacturer’s protocol (Abcam, Cambridge, UK). Briefly, the cells were grown overnight in a 96-well plate in a growth medium at a concentration of 1 × 10^4^ cells/90 µL per well. The plate was centrifuged at 800 rpm for 2 min. The medium was removed from wells and 100 µL/well of OH580 Stain Working Solution was added to each well of the plate followed by incubation at 37 °C for one hour. The cells were infected with stress-treated bacteria in DMEM and incubated at 37 °C for 2 h in the dark. The cells were then washed thrice with Dulbecco’s Phosphate-Buffered Saline (DPBS). One hundred microliters of Assay Buffer was added to each well and fluorescence was recorded at Ex/Em = 540/590 nm with bottom read mode. HeLa and HepG2 cells treated with Fenton reaction (10 µM CuCl_2_ and 100 µM H_2_O_2_) at 37 °C for 1 h served as a positive control, whereas non-treated cells served as a negative control.

### 4.6. Effect on Actin during ST Interaction for Invasion

To evaluate the changes in the actin cytoskeleton during host–ST interactions, actin was stained using fluorescent dye 594-I Phalloidin following the manufacturer’s instructions (Abnova, Taipei, Taiwan). Briefly, the cells were plated and infected as in the gentamicin protection assay. After 60 min, the plates were thoroughly washed twice in PBS. The cells were then fixed with 4.0% formaldehyde for 15 min at room temperature and permeabilized using 0.1% Triton X-100 after washing with PBS. Then, 100 µL of phalloidin conjugate working solution was added to the fixed cells and incubated for 30 min at room temperature. The nuclei were stained using DAPI (Sigma, St. Louise, MO, USA). After gently rinsing cells with PBS three times, microscopic observations were conducted using a fluorescent microscope (Zeiss, Oberkochen, Germany). Accumulation of phalloidin staining at the site of interaction between host cell bacteria was considered to indicate the presence of polymerized actin.

### 4.7. Localization of ST in the HeLa and HepG2 Cells

Cells were grown on coverslips and infected with ST (JOL 401, JOL 912, and JOL 1800). After 30 min, the cells were washed thoroughly with PBS to remove extracellular bacteria, and a medium containing gentamicin was added. After, 2 hpi cells were mounted on the glass slide using glycerol (90%) in PBS. Cells were observed under the confocal microscope equipped with a 100× oil immersion objective (Zeiss, Germany). The location of the GFP plasmid-carrying bacteria was used for infection in this study. The results were obtained from three different replicates.

### 4.8. Expression of Cytokines, Cyclin, and Apoptosis Genes

Expression of selected cytokine and chemokine genes NF-kB, IFN-γ, CXCL 8, and CXCL 10 was assessed in HepG2 after 12 h of incubation at a multiplicity of infection 100. Time course experiments were carried out for cyclins (D1 and B1) and caspase 3 and 7 gene expression in HeLa and HepG2 following infection with ST WT and mutants. For this, HeLa and HepG2 with bacterial infection were kept for 12 h and used for total RNA isolation. RNA isolated from 0 h cells were considered as the control.

### 4.9. Real-Time Quantitative PCR (qPCR) Analysis

Total RNA from HeLa and HepG2 was extracted by TRIzol method (Chomczynski and Sacchi, 1987) [60] and concentration was determined using a biophotometer. The total RNA was treated with RNase-free DNase I (Thermo Fisher Scientific, Waltham, MA, USA) to remove genomic DNA contamination. Total RNA in each group was reverse transcribed into first strand cDNA in a 10 μL reaction using ReverTra Ace^®^ qPCR RT kit (Toyobo, Tokyo, Japan) according to the manufacturer’s instructions. The relative expression of cytokines, cyclins, and caspase genes in HeLa and HepG2 cells were quantified and compared using a real-time PCR instrument equipped with StepOne software V2.2.2 (Applied Biosystems, Foster City, CA, USA). Glyceraldehyde 3-phosphate dehydrogenase (GAPDH) was used as a housekeeping gene. The reaction mixture (10 μL) consisted of 2 μL cDNA, 5 μL 2x SYBR green master mix (Thermo Fisher Scientific Inc., USA), 0.5 μmol/L primer pairs, and 2 μL PCR-grade water (Sigma-Aldrich, St. Louis, MO, USA). A no-template control reaction was included in each assay. The primers used in this study were designed based on the prior sequence information and basic local alignment search tool (BLAST) analysis against the *Homo sapiens* genome (Table 2). Relative fold change of a target gene was carried out by comparing the expression level of the reference gene (GAPDH) using the standard curve method (Pfaffl, 2001) [61].

### 4.10. Cell Cycle Analysis by Flow Cytometer

HeLa and HepG2 cells were seeded DMEM complete medium (10% FBS, 1% antibiotics) in 12 well plates at 2 × 10^5^ cells/well and incubated at 37 °C in a 5% CO^2^ atmosphere. When the cells were 80% confluence, cells were infected with JOL 401, JOL 912, or JOL 1800 at 40 MOI. Infection was carried out for 2.5 h and cells were washed with PBS two times and replaced with fresh medium containing 100 μg/mL gentamycin. After 2.5 h incubation with gentamycin to eliminate non-infected bacteria, the cell medium was replaced with fresh medium containing 10 μg/mL gentamycin and further incubated for 12 h. After, incubation cells were harvested by flushing using a pipette, and cells were fixed by gradually adding 500 μL of 100% ice-cold ethanol, gently mixed by pipetting in and out at least ten times, and then kept on ice for 15 min. Cells were centrifuged at 500× *g* for 10 min and re-suspended in 150 μL of propidium iodide (5 μg/mL) and RNAses (1 μg/mL). Cells were gently mixed and incubated at 37 °C for 40 min. Cells were collected by spinning and washed once with PBS and re-suspended in 500 μL Macsquant running buffer for flow cytometry analysis. After applying appropriate gating procedures, flow cytometer analysis of cell cycle kinetics was carried out using a Macsquant Flow Cytometer (Miltenyi, Bergisch, Gladbach, Germany).

### 4.11. Statistical Analysis

All the experiments were replicated three times. Results were expressed as the means ± SEM. A difference with a value of *p* < 0.05 was considered statistically significant. Data were analyzed by analysis of variance (ANOVA) and statistical differences between various treatment group means were determined by least square difference using the Statistical Product and Service Solutions, Version 17.0.1 software (SPSS Inc., Chicago, IL, USA).

## Figures and Tables

**Table 1:** *Salmonella* Typhimurium wild type and mutants used in this study.

Bacteria	Features
JOL 401	*Salmonella* Typhimurium wild type, SPI-*1 invAE*^+^, *hilA*^+^, *avr*^+^; SPI-2, amino acid permease; SPI-3, *mgtC*^+^; SPI4, ABC transporter; SPI5, *pipB*^+^
JOL 911	JOL 401 ∆*lon*, ∆*cpxR*; smooth ST strain
JOL 912	JOL 401 ∆*lon*, ∆*cpxR*, Δ*asd*; smooth ST strain
JOL 1800	JOL 912 ∆*rfaL*, O-antigen deficient strain, improved bacterial delivery vector; rough ST strain

**Table 2:** Primer sequences of each gene target were analyzed in HeLa and HepG2 cells by quantitative real-time PCR.

Gene	Primer	Sequence (5′ → 3′)
Nuclear factor-κB	Forward	TGGGACCAGCAAAGGTTATT
	Reverse	GATCCCATCCTCACAGTGTTT
Interferon-γ	Forward	ATGTCCAACGCAAAGCAATAC
	Reverse	ACCTCGAAACAGCATCTGAC
Cyclin D1	Forward	CATCTACACCGACAACTCCATC
	Reverse	TCTGGCATTTTGGAGAGGAAG
Cyclin B1	Forward	GGCTTTCTCTGATGTAATTCTTGC
	Reverse	GTATTTTGGTCTGACTGCTTGC
Cyclin B2	Forward	CCTCCCTTTTCAGTCCGC
	Reverse	CTCCTGTGTCAATATTCTCCAAATC
Caspase 3	Forward	AATGGACCTGTTGACCTGAAA
	Reverse	CACGGCAGGCCTGAATAA
Caspase 7	Forward	CTGACTTCCTCTTCGCCTATTC
	Reverse	TCTGCATGATTTCCAGGTCTT
CXCL 8	Forward	GAGAGTGATTGAGAGTGGACCAC
	Reverse	CACAACCCTCTGCACCCAGTTT
CXCL 10	Forward	GGTGAGAAGAGATGTCTGAATCC
	Reverse	GTCCATCCTTGGAAGCACTGCA
GAPDH	Forward	CCCTTCATTGACCTCAACTACA
	Reverse	ATGACAAGCTTCCCGTTCTC

## Data Availability

Data are available from the authors upon reasonable request.
